# Randomized controlled clinical trial on the efficacy of a novel antimicrobial chewing gum in reducing plaque and gingivitis in adolescent orthodontic patients

**DOI:** 10.1007/s00784-024-05669-4

**Published:** 2024-04-25

**Authors:** Johanna Weber, Konstantin J. Scholz, Isabelle M. Schenke, Florian Pfab, Fabian Cieplik, Karl-Anton Hiller, Wolfgang Buchalla, Camilla Sahm, Christian Kirschneck, Eva Paddenberg-Schubert

**Affiliations:** 1 Department of Conservative Dentistry and Periodontology, University Hospital Regensburg, Regensburg, Germany; 2 Department of Orthodontics, University Hospital Regensburg, Regensburg, Germany; 3 Department of Operative Dentistry and Periodontology, Center for Dental Medicine, Medical Center, Faculty of Medicine, University of Freiburg, University of Freiburg, Freiburg, Germany; 4 Private pediatric dental practice, Regensburg, Germany; 5 Department of Dermatology and Allergology, Technische Universität München, Munich, Germany; 6 Medical Department Eintracht, Frankfurt Soccer AG, Frankfurt, Germany; 7 Department of Orthodontics, University of Bonn, Bonn, Germany

**Keywords:** Chewing gum, Essential oil, Plaque accumulation, Gingivitis, Orthodontic treatment

## Abstract

**Objectives:**

Chewing gums containing antiseptics or other antimicrobial substances may be effective in reducing plaque and gingivitis. Therefore, the aim of this randomized placebo-controlled clinical trial was to investigate the efficacy of a novel antimicrobial chewing gum containing essential oils (cinnamon, lemon, peppermint) and extracts on reduction of dental plaque and gingivitis as well as on oral health-related quality of life (OHRQoL) in adolescent orthodontic patients.

**Materials:**

52 patients (11-22 years of age) were randomly assigned to use a test chewing gum (COVIDGUM, Clevergum) or a commercially available control chewing gum over a period of 10 days. Approximal plaque index (API), papillary bleeding index (PBI) and an OHRQoL questionnaire for children (COHIP-G19) were assessed at baseline (BL), after 10 days (10d) and 30 days (30d). In addition, oral health and oral hygiene related questions of the COHIP-G19 questionnaire were evaluated separately in subscales at each timepoint. Data were analyzed using non-parametrical statistical procedures (α = 0.05).

**Results:**

API and PBI decreased significantly over time from BL to 10d and from BL to 30d in both groups, without significant differences between the groups. In both groups, the COHIP-G19 score, oral health subscale and oral hygiene subscale decreased significantly over time. Regarding the oral hygiene subscale, the test group showed significantly better scores at 30d (*p *= 0.011).

**Conclusion:**

Both chewing gums performed similarly effective in terms of reducing plaque accumulation and gingival inflammation and improving OHRQoL.

**Clinical relevance:**

Chewing gums without antimicrobial ingredients may be sufficient to decrease plaque accumulation and gingival inflammation.

## Introduction

The demand for orthodontic treatment with fixed or removable appliances is increasing for correcting misaligned teeth or jaws and treating functional disorders in adolescents and adults. Accordingly, data from the 6^th^ German Oral Health Study (DMS • 6) shows that around 40% of children in Germany are in need of orthodontic treatment [1]. During orthodontic treatment, particularly with fixed orthodontic appliances, the ability to maintain oral hygiene is restricted [2]. Accordingly, the environmental conditions in the oral cavity change, leading to an increased accumulation of dental biofilm, especially in the areas around the brackets where sufficient mechanical plaque removal is difficult to be achieved [3, 4]. In most cases, regular mechanical cleaning using toothbrushes is not sufficient to remove the biofilm adequately [5]. Therefore, patients with fixed orthodontic appliances often develop gingivitis [6], with adolescents being particularly affected during puberty [7]. For instance, Levin et al. showed that the gingival index as well as bleeding on probing were significantly increased in orthodontic patients between 18 and 26 years compared to patients not undergoing orthodontic treatment [8].

To counteract the development of gingivitis, antiseptic mouthwashes containing cationic biocides such as chlorhexidine digluconate (CHX) or cetylpyridinium chloride (CPC) have been shown effective in the prevention of biofilm accumulation and gingival inflammation [9] and as preprocedural mouthwashes before dental treatments [10]. CHX mouthwashes have also been shown to successfully control gingival inflammation in orthodontic patients [11]. Despite such positive effects of antiseptic mouthwashes, potential risks associated with antiseptic resistance should not be ignored given their frequent use in dentistry [12, 13]. For instance, clinical oral isolates of early colonizers in dental plaque revealed phenotypic adaptation to CHX and CPC upon multiple exposure to subinhibitory concentrations *in vitro* [14], which may be due to transcriptomic changes and up-regulation of pathways associated with antibiotic resistance [15]. In addition, CHX and CPC have strong effects on the microbial composition of biofilms when frequently treated with these agents [16–19]. Furthermore, it is also well known that the frequent use of products containing CHX can lead to teeth and tongue staining, mucosal irritation and burning mouth [20, 21].

Besides antiseptic mouthwashes, products containing essential oils have also shown to be effective for the management of gingivitis in orthodontic patients [22]. The six-month study by Cortelli et al. showed that a mouthwash with essential oils (Listerine^®^, Johnson & Johnson, New Brunswick, NJ, USA) applied twice daily led to a reduction in gingivitis, bleeding and plaque accumulation compared with a CPC and a placebo rinseat all post-baseline time-points [23].

Since chewing gums usually stay in the mouth much longer than mouthwashes, allowing its active ingredients to be effective longer [24], they could be a good alternative to reduce dental plaque and gingivitis in patients undergoing orthodontic treatment. The effects of a chewing gum containing CHX have been investigated in orthodontic patients regarding reductions of plaque levels and gingival bleeding [21]. There were no significant differences between the CHX and the placebo gum at either examination timepoint but both chewing gums led to significant decreases of bleeding on probing along with partly significant decreases of plaque levels over time [21].

On the other hand, chewing gums containing mastic or polyphenols such as quercetin showed promising results on temporary reduction of bacterial loads in the oral cavity when compared to a placebo gum [24–26]. However, clinical data on potential reductions of biofilm accumulation and gingival inflammation are missing yet for chewing gums containing essential oils. Recently, an antimicrobial chewing gum (COVIDGUM, Clevergum GmbH, Munich, Germany) containing essential oils (cinnamon, lemon, peppermint) and extracts (ginger, ginseng, quercetin, and spermidine) was marketed and has been shown to significantly reduce the viral load of SARS-CoV-2 in exhalative air [27].

The aim of this randomized controlled double-blinded clinical trial was to investigate the efficacy of COVIDGUM on reduction of dental plaque and gingivitis as well was oral health-related quality of life in adolescent orthodontic patients compared to a control chewing gum in addition to regular mechanical oral hygiene. The null hypothesis tested was that there were no differences in clinical parameters (plaque levels and gingival inflammation) as well as in oral health-related quality of life of gingivitis patients over time and between the test and control chewing gums.

## Material & Methods

### Study design & ethical considerations

The present study is a prospective randomized controlled double-blinded clinical trial comparing a novel antimicrobial chewing gum (COVIDGUM, CleverGum GmbH, Grünwald, Germany) containing essential oils and extracts as the test group and a commercially available chewing gum (Airwaves Cool Cassis, Mars GmbH, Verden, Germany) as the control group for their efficacy in reducing plaque and gingivitis in adolescent patients with orthodontic appliances. Table 1 shows the ingredients of both chewing gums.

**Table 1:** Ingredients of both chewing gums

Group	Chewing gum	Manufacturer	Ingredients
Test	CovidGum	CleverGum GmbH (Grünwald, Germany)	Essential oils (cinnamon, peppermint, lemon), ginger, ginseng, zinc, quercetin, spermidine, vitamin D3, xylitol, sucralose
Control	Airwaves Cool Cassis	Mars GmbH (Verden, Germany)	Sweetener sorbitol, isomalt, malitol syrup, aspartame, barelyasse, flavors, humectant glycerin, thickener gum arabic, emulsifier soy lecithin, mannitol, aesulfame K, sucralose, coating agent carnauba wax, colorant E163, antioxidant BHA

As primary outcome, plaque accumulation and gingival inflammation were assessed by means of the approximal plaque index (API) according to Lange et al. [28] and the papilla bleeding index (PBI) according to Saxer and Mühlemann [29]. As secondary outcome, the oral health-related quality of life was measured by means of a questionnaire containing 19 questions based on the German version of the Child Oral Health Impact Profile (COHIP-G19) [30].

The study design was approved by the Internal Review Board of the University of Regensburg (Ref. 22-2952-101) in accordance with the 1964 Declaration of Helsinki and its subsequent amendments or comparable ethical standards and followed the requirements of the CONSORT 2010 Statement [31]. The study was prospectively registered in the German Clinical Trials Registry (Ref. DRKS00030056).

### Patient selection

Patients were recruited from the patient pools of multiple study centers located in Germany, which included the Department of Orthodontics at the University Hospital Regensburg, private orthodontic practices in Cham and Paderborn, and a private pediatric dental practice in Regensburg. For inclusion, patients had to be between 11 to 22 years of age and under orthodontic treatment with fixed or removable appliances. Furthermore, they had to exhibit non-sufficient oral hygiene as indicated by a full-mouth API > 40%. The exclusion criteria included serious general diseases such as diabetes mellitus, tumor diseases, rheumatoid arthritis, known allergies or intolerances to one or more of the ingredients of the two chewing gums, the use of antibiotics within the last 3 months and the need for periodontal treatment as recorded by the periodontal screening index to prevent distortion of the data. Written informed consent was obtained from all included patients and their legal representatives.

### Clinical examinations

The study was divided into an initial examination, followed by the application phase of the chewing gum, and two follow-up examinations after different periods of time. At the baseline (BL) examination, a thorough dental examination was carried out, in which the API according to Lange et al. [28] and PBI according to Saxer and Mühlemann [29] were collected. Patients were also asked to complete a COHIP-G19 questionnaire [30]. Finally, the patients were randomly assigned to the test or control group using a computer-generated randomization table. Patients were blinded to the respective chewing gum, which was ensured by identical packaging, and clinical follow-up examinations were performed by blinded examiners.

In the subsequent application phase, the patients used the assigned gum for 15 minutes four times a day for a period of 10 days and were instructed to apply their usual oral hygiene routine. No additional detailed oral hygiene instructions were given. The first follow-up examination was performed 10 ± 1 days after baseline (10d). Again, API and PBI were measured and the COHIP-G19 questionnaire was filled out. 30 ± 2 days after baseline, the second and final follow-up examination were carried out (30d), in which API and PBI were measured and the COHIP-G19 questionnaire was filled out again. If gingivitis persisted, the patients received a detailed instruction to improve their oral hygiene as well as a professional tooth cleaning.

## Assessment of oral health‑related quality of life

The German version of the Child Oral Health Impact Profile (COHIP-G19) by Sierwald et al. was used to assess the oral health-related quality of life (OHRQoL) of the participating children and adolescents [30]. The German COHIP-G19 is a short version with only 19 questions of the COHIP questionnaire, which originally contained 34 items. Furthermore, it can be divided into three subscales: the oral health subscale (Questions 1- 5), the functional well-being subscale (Questions 9, 13, 17, 18), and the social/emotional, school and self-image subscale (Questions 6, 7, 8, 10, 11, 12, 14, 15, 16, 19) [32]. Each of the 19 questions was answered on a Likert scale (0 = never, 1 = almost never, 2 = sometimes, 3 = quite often and 4 = almost always) in terms of how often the positive or negative impact was experienced in the last three months. The COHIP-G19 enables the calculation of summary values for the overall instrument and for subscales. The summary scores can be highest at 76 and lowest at 0 [30], whereby smaller values represent a lower OHRQoL [32]. In addition, a “oral hygiene” subscale (Questions 2, 4, 5) was newly defined from questions 2, 4, and 5 that were considered relevant to the topic of the present study and addressed discolorations (question 2), bad breath (question 4) and bleeding gums (question 5). The highest possible value that could be reached with this new subscale was 12. Patients were asked to fill out the COHIP-G19 questionnaire at BL, 10d and 30d.

## Data analysis

Data analysis was performed using SPSS for Windows, v. 26 (SPSS Inc., Chicago, IL, USA). The patient represented the statistical unit. Data are reported as median values (with first and third quartiles) or proportions (numbers of patients), respectively, and were analyzed applying non-parametric statistical procedures at a significance level α = 0.05. Mann-Whitney *U* tests or χ^2^ tests were used for pairwise comparisons between test and control groups, while Wilcoxon signed-rank tests were used for pairwise comparisons within one group over time.

## Results

### Patient characteristics

**Fig. 1:**
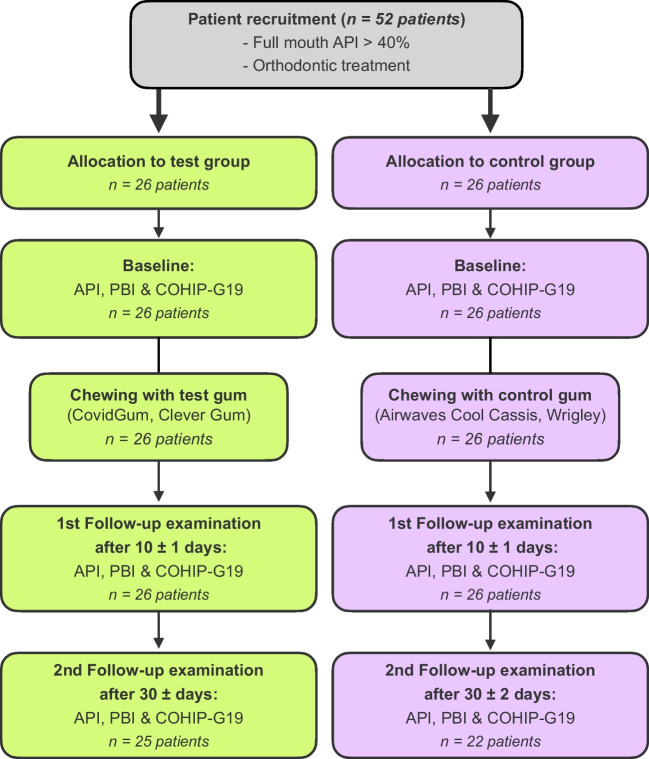
Flow of patients through the stages of this study

52 patients were included in this prospective randomized controlled clinical trial, of whom 26 were randomly assigned to the test group and 26 to the control group. All of them underwent orthodontic treatment with fixed appliances, as those with removable appliances did not present a full-mouth API > 40%. Figure 1 shows the CONSORT flow of patients for this study, and Table 2 summarizes the patient characteristics of all individual patients included in this study. The median age of the participants was 14.5 years in the test group and 14.4 in the control group. The proportions of female to male participants were 14 to 12 in the test and 13 to 13 in the control group, respectively. There were no significant differences between test and placebo groups regarding patient characteristics.

**Table 2:** Patient characteristics

Variables		Test group (*n = 26)*	Control group (*n = 26)*	Significant differences
Age^a^	[years]	14.5 (13.1; 15.8)	14.4 (12.7; 17.3)	–
Sex^b^	female	53.8% (14)	50% (13)	–
	male	46.2% (12)	50% (13)	–

Depiction of medians^a^ (first quartile; third quartile) or relative proportions^b^ (numbers of patients) and statistically significant differences from pairwise comparisons (Mann–Whitney U or χ^2^ -tests, respectively; α = 0.05)

*p*-value, significant (*p* ≤ 0.05); –, not significant (*p* > 0.05)

### Clinical parameters

All clinical parameters are shown in Table 3. At BL, the median API was measured 81.4% in the test group and 80.4% in the control group. At the first follow-up after 10d, the measured median API decreased to 68.0% in the test and to 66.7% in the control group. At the last follow-up at 30d, the median API was 66.6% in the test and 64.9% in the control group. While there were no significant differences found between the groups, the decreases in API were significant for both groups between BL and 10d (test: *p *< 0.001; control: *p *= 0.011) and BL and 30d (test: *p *< 0.001; control: *p *= 0.023).

**Table 3:** Clinical parameters

Clinical parameter	Timepoint	Test group	Control group	Significant differences			
				between groups	over time		
					BL vs. 10d	10d vs. 30d	BL vs. 30d
API	BL	81.4 (73.1; 89.2)	80.4 (62.9; 86.7)	*–*	Test: *p *< 0.001 Control: *p *= 0.011	Test: *–* Control: *–*	Test: *p *< 0.001 Control: *p *= 0.023
	10d	68.0 (62.9; 77.9)	66.7 (45.9; 83.8)	*–*			
	30d	66.6 (56.9; 73.3)	64.9 (46.6; 86.6)	*–*			
PBI	BL	47.7 (26.6; 72.0)	40.0 (26.3; 69.6)	*–*	Test: *p *< 0.001 Control: *p *= 0.021	Test: *–* Control: *–*	Test: *p *< 0.001 Control: *p *= 0.039
	10d	29.6 (14.6; 62.8)	29.7 (15.0; 56.4)	*–*			
	30d	30.8 (17.5; 54.4)	30.7 (16.3; 68.0)	*–*			

Depiction of medians (first quartile; third quartile) and statistically significant differences from pairwise comparisons between groups (Mann–Whitney *U* tests) or over time (Wilcoxon signed-rank tests), respectively (α = 0.05)

*p*-value, significant (*p* ≤ 0.05); –, not significant (*p* > 0.05)

In both groups, there was a decrease in median PBI from BL to 10d, i.e. from 47.7% to 29.6% in the test group and from 40.0% to 29.7% in the control group. At 30d, however, PBI rose again slightly to 30.8% in the test group and 30.7% in the control group. Despite no significant intergroup differences, there were significant changes of the PBI over time between BL and 10d (test: *p *< 0.001; control: *p *= 0.021) and BL and 30d (test: *p *< 0.001; control: *p *= 0.039) in the control group. Within the time period of the intervention and follow-up, no damaging effects on the fixed appliances, such as bracket loss, could be observed.

### Oral health-related quality of life

The evaluation of the OHRQoL by means of the COHIP-G19 is presented in Table 4. Patients had a median total COHIP-G19 score at BL of 16.5 in the test group and 17.0 in the control group. The total score decreased significantly in both groups over time. In the test group, there were significant decreases from BL to 10d (*p *< 0.001), from BL to 30d (*p *< 0.001) and from 10d to 30d (*p *= 0.048). Similar results were observed in the control group (BL vs. 10d: *p *= 0.002; BL vs. 30d: *p *< 0.001), but there was no significant change between 10d and 30d. No significant differences were found between the two groups at any timepoint.

**Table 4:** COHIP-19 summary score and subscales.

COHIP-G19	Timepoint	Test group	Control group	Significant differences			
				between groups	over time		
					BL vs. 10d	10d vs. 30d	BL vs. 30d
COHIP-G19 summary scale (max. score: 76)	BL	16.5 (11.0; 22.3)	17.0 (10.8; 20.0)	*–*	Test: *p *< 0.001 Control: *p *= 0.002	Test: *p *= 0.048 Control: *–*	Test: *p *< 0.001 Control: *p *< 0.001
	10d	12.5 (8.8; 19.5)	11.0 (9.0; 16.3)	*–*			
	30d	9.0 (5.5; 15.5)	10.0 (8.0; 14.3)	*–*			
COHIP-G19 oral health subscale (max. score: 20)	BL	6.0 (5.0; 7.0)	7.0 (4.0; 9.3)	*–*	Test: *p *= 0.001 Control: *p *= 0.001	Test: *–* Control: *–*	Test: *p *= 0.002 Control: *p *= 0.005
	10d	4.5 (2.8; 6.3)	4.0 (3.0; 7.3)	*–*			
	30d	3.0 (2.0; 6.0)	4.5 (3.8; 8.0)	*–*			
COHIP-G19 functional well-being subscale (max. score: 16)	BL	4.0 (3.0; 6.0)	4.0 (2.0; 5.0)	*–*	Test: *–* Control: *–*	Test: *p *= 0.042 Control: *–*	Test: *–* Control: *–*
	10d	4.0 (2.0; 6.0)	3.0 (2.0; 4.3)	*–*			
	30d	3.0 (2.0; 5.0)	2.5 (1.0; 4.0)	*–*			
COHIP-G19 socio-emotional, well-being subscale (max. score: 40)	BL	5.5 (4.0; 10.8)	5.5 (2.8; 7.0)	*–*	Test: *p *= 0.019 Control: -	Test: *p *= 0.001 Control: *p *= 0.004	Test: - Control *p *= 0.022
	10d	4.5 (2.0; 9.3)	4.0 (2.0; 7.0)	*–*			
	30d	4.0 (2.0; 7.0)	3.0 (1.8; 5.0)	*–*			
COHIP-G19 oral hygiene subscale (max. score: 12)	BL	4.0 (2.0; 5.3)	5.0 (3.0; 6.0)	*–*	Test: *p *= 0.004 Control: *p *= 0.003	Test: *–* Control: *–*	Test: *p *= 0.002 Control: *p *= 0.022
	10d	3.0 (1.0; 4.0)	3.0 (2.0; 4.0)	*–*			
	30d	2.0 (1.0; 3.0)	3.0 (2.3; 4.0)	*p *= 0.011			

Depiction of medians (first quartile; third quartile) and statistically significant differences from pairwise comparisons between groups (Mann–Whitney *U* tests) or over time (Wilcoxon signed-rank tests), respectively (α = 0.05)

*p*-value, significant (*p* ≤ 0.05); –, not significant (*p* > 0.05)

The oral health subscale also changed in both groups from BL to 10d (test: *p *= 0.001; control: *p *= 0.001) and from BL to 30d (test: *p *= 0.002; control: *p *= 0.005).

Regarding the functional-well-being subscale, a significant difference could only be observed in the test group from 10d to 30d (*p *= 0.042).

When looking at the socio-emotional, well-being subscale, in the control group there was a significant decrease from BL to 10d (*p *= 0.019) as well as from 10d to 30d (*p *= 0.001), and in the test group from 10d to 30d (*p *= 0.004) and from BL to 30d (*p *= 0.022). No significant difference was found between the groups at any timepoint.

Concerning the oral hygiene subscale, which was first described in this study, patients in the test group showed a significantly lower score compared to the control group at 30d (*p *= 0.011). A significant improvement in the oral hygiene subscale was also observed within the groups from BL to 10d (test: *p *= 0.004; control: *p *= 0.003) and from BL to 30d (test: *p *= 0.002; control: *p *= 0.022).

## Discussion

The use of chewing gums containing antiseptics such as CHX and CPC for decreasing plaque accumulation and gingival bleeding has been investigated in several clinical studies [21, 33, 34], but data on the use of chewing gums containing essential oils is still lacking. Thus, the aim of this study was to evaluate the efficacy of a novel antimicrobial chewing gum with essential oils in improving clinical parameters and oral health-related quality of life in orthodontic patients with gingivitis. This study had a randomized, double-blinded and parallel design with 26 patients in each group according to previous studies [21, 33, 34]. The distribution of the two study groups did not significantly differ regarding the factors gender and age.

In the study of Cosyn and Verelst [21], where patients chewed either CHX-containing gum or control gum for 10 minutes twice a day over a period of 3 months, no significant differences regarding plaque level and gingival inflammation were found between the two groups. However, significant improvements in gingival bleeding on probing over time were observed for both groups and in plaque levels for the control group [21]. Simons et al. investigated the efficacy of chewing gum containing CHX and xylitol in terms of plaque and gingival indices in elderly occupants of residential homes by comparing it to a xylitol chewing gum and a control group which did not use any chewing gum [33, 34]. It was found that both plaque and gingival indices decreased significantly in the group with a gum containing both CHX and xylitol, while in the xylitol gum group only the plaque index decreased significantly, and no significant change was observed in the control group [33, 34].

Many studies have already shown that chewing gum increased the salivary flow rate [35–37]. In the long term, this may be associated with a decreased plaque formation, as shown in the studies mentioned above [21, 33]. In the present study, API of both the test group and the control group decreased significantly over time from BL to 10d and from BL to 30d. However, no significant difference could be demonstrated between the groups, which supports the hypothesis that chewing gum alone without an active ingredient could stimulate salivary flow and thus decrease the plaque index.

Previous studies have shown that mouthwashes containing CHX and essential oils could decrease gingival inflammation [10, 38–41]. Accordingly, our results showed a significant decrease in PBI at 10d and at 30d compared to BL in both groups. This is in line with the other studies mentioned above [21, 33, 34]. However, no significant difference between the groups could be demonstrated here. This could be explained by the fact that the 10-day period of using the test chewing gum may have been too short to detect differences in the PBI among the groups since the PBI is a parameter for monitoring medium- to long-term oral hygiene. Accordingly, Simons et al. who investigated the effects of chewing gum over 12 months, found significantly better reduction of gingival bleeding for the group using the CHX-containing chewing gum [33, 34].

Another limitation of the present study was that no intermediate clinical controls were carried out during the application period and thus a correct application, 4 times a day for 10 minutes, could not be guaranteed by the examiners. However, the clinical study by Ainamo et al. showed that the release of antibacterial substances in CHX chewing gum is time dependent [42]. This could also apply to the essential oils in the test chewing gum in this study, although this aspect was not examined here.

Oral health-related quality of life was assessed with the COHIP-G19. This German short version of the questionnaire was first described by Sierwald et al. [30] and has been used in a few studies so far. The instrument was shown to have psychometric properties to measure oral health-related quality of life in children and adolescents [30, 43, 44]. While another German questionnaire, the CPQ G_11-14_, is only approved for children aged 11 to 14 years, the COHIP-G19 covers an age range from 7 to 18 years [45, 46]. Long survey instruments, such as the CPQ G_11-14_ with its 37 questions, can also be burdensome for participants, especially children and adolescents, and require more effort and time, while shorter questionnaires are easier to score and interpret and may therefore be more useful for clinicians [32, 47].

When looking at the OHRQoL, a decrease in the total score was observed in both groups. However, it is noteworthy that there was a significant difference between the groups in the “oral hygiene” subscale at 30d. With these results, one could conclude that chewing gum with essential oils as an ingredient could have positive effects on oral hygiene-related quality of life. Not only did the oral hygiene subscale decrease significantly in the test group, but a closer look at the results for question 4 with focus on bad breath, showed that the score improved significantly in the test group from BL to 10d (*p *= 0.018) and from BL to 30d (*p *= 0.024). There was no significant difference between the two groups at any timepoint. The reason for this could be the antibacterial effects of cinnamon oil and especially cinnamaldehyde, which may be particularly effective against volatile sulphur compounds (VSC)-producing bacteria that are known to be partly responsible for bad breath [48]. For example, Zhu et al. investigated the short-term bacteria killing of a cinnamon chewing gum in their double-blinded, cross-over clinical study. A chewing gum containing natural cinnamon flavor and cinnamaldehyde was compared to one with natural cinnamon flavor but without cinnamaldehyde. Both chewing gums showed a significant reduction in H_2_S-producing bacteria [49] However, these results should be treated with caution, as the COHIP response is based on subjective perceptions.

The null hypothesis of the present study could only be rejected in part. No significant differences were found between the two chewing gums, and the use of both led to a significant improvement in the clinical parameters API and PBI over time. Only the hygiene subscale of the COHIP questionnaire showed a significant difference between the two chewing gums, with the test gum achieving better results and reducing the score of this subscale over time.

The results indicate that further studies with longer application periods and more frequent follow-up examinations are necessary to provide more reliable data for the recommendation of chewing gum. This will also help to analyze potential damaging effects of the chewing gums, such as bracket loss, on the fixed appliances in the long run. Furthermore, measurement of halitosis should also be considered to obtain clinical evidence of improvement in bad breath.

## Conclusion

This study showed that in orthodontic patients both, a chewing gum containing essential oils and a commercially available control chewing gum, significantly reduced plaque growth and gingivitis. Both also had a positive effect on oral health-related quality of life, with a tendency for better performance of the test gum. Chewing gum as a supplement to regular oral hygiene could show positive effects, especially in patients who have difficulties with oral hygiene at home.

## Data Availability

The data that support the findings of this study are available from the corresponding author upon reasonable request.
